# Nano-Scale Positioning Design with Piezoelectric Materials

**DOI:** 10.3390/mi8120360

**Published:** 2017-12-12

**Authors:** Yung Yue Chen, Yung Hsiang Chen, Chiung Yau Huang

**Affiliations:** 1 Department of Systems and Naval Mechatronics Engineering of National Cheng Kung University, Tainan 701, Taiwan; 2 Department of Mechanical Engineering of National Pingtung University of Science and Technology, Pingtung 91201, Taiwan; yhchen@mail.npust.edu.tw; 3 Hsien Chi Primary School, Pingtung 93245, Taiwan; kikihcyychen@yahoo.com.tw

**Keywords:** piezoelectric material, Bouc–Wen model, hysteresis effect, nano-scale positioning design, nonlinear control

## Abstract

Piezoelectric materials naturally possess high potential to deliver nano-scale positioning resolution; hence, they are adopted in a variety of engineering applications widely. Unfortunately, unacceptable positioning errors always appear because of the natural hysteresis effect of the piezoelectric materials. This natural property must be mitigated in practical applications. For solving this drawback, a nonlinear positioning design is proposed in this article. This nonlinear positioning design of piezoelectric materials is realized by the following four steps: 1. The famous Bouc–Wen model is utilized to present the input and output behaviors of piezoelectric materials; 2. System parameters of the Bouc–Wen model that describe the characteristics of piezoelectric materials are simultaneously identified with the particle swam optimization method; 3. Stability verification for the identified Bouc–Wen model; 4. A nonlinear feedback linearization control design is derived for the nano-scale positioning design of the piezoelectric material, mathematically. One important contribution of this investigation is that the positioning error between the output displacement of the controlled piezoelectric materials and the desired trajectory in nano-scale level can be proven to converge to zero asymptotically, under the effect of the hysteresis.

## 1. Introduction

The ultrahigh precision motion of a nano-scale workspace is required for the development of precision industries. In the past decades, piezoelectric actuators are widely used in the applications of micro-positioning, due to their advantages of micro/nanometer positioning resolution, a fast response, large force, and so on. However, piezoelectric based actuators naturally exhibit strong hysteresis nonlinearity. This hysteresis nonlinearity will seriously fade the positioning accuracy of the piezoelectric actuators, and let the piezoelectric actuators be unstable. In several physical systems, such as optic, biology, magnetism, mechanic, etc., hysteresis phenomena always exist. The output of a system with the hysteresis effect is independent of the input. As the input alternates between the increasing and the decreasing condition, the response curve diverges from its original path and draws a new curve, i.e., output of the piezoelectric material relies not only on the current input, but also depends on its history due to a memory effect that exists in the piezoelectric material. Based on the depicted reason, to approach a high accurate positioning design, the remedy must be to reduce the effect of hysteresis of the piezoelectric materials. An effective solution for tackling the positioning problem of piezoelectric materials is to mathematically model the hysteresis behavior and cancel it. There are three categories of models adopted to present the hysteresis property of piezoelectric materials in the existing investigations. The first one is the physical modeling theory, which is represented by the Jiles–Atherton model [1] and Duhem model [2]. It is one kind of dynamic model described by a set of high order differential equations. The second one is based on the operator modelling theory, which is proposed by authors Preisach [3], Krasnoselskii–Pokrovskii (KP) [4], Prandtl–Ishlinskii (PI) [5], etc., but the mathematical formulations of these models are too complicated. For the purpose of effectively modeling the behavior of the piezoelectric materials with a simple form, the Bouc–Wen model [6,7], with a set of first order differential equations, was proposed because this model has the advantages of computational simplicity and practical similarity. Generally speaking, for nano-scale positioning designs, differential equation models including simple Dahl model, Bouc–Wen model, backlash-like model, Maxwell model, approximated polynomial model, and so on, are preferred, due to the fact that many control methods are already well-developed for them. In order to achieve a high-accuracy positioning performance, a thorough analysis of the hysteresis nonlinearity, and the appropriate compensation design for the piezoelectric material, are necessary. Typically, the most direct way for mitigating the effects of hysteresis is to use the inverse operator. This is a method with creating a compensator based on an inversion of the hysteresis model, such as Bouc–Wen model or others. However, the inverse operator is only applicable with some particular input signal. In practical implementation, the inverse operator is very complicated, and even impossible to be realized, due to multi-values and the non-smoothness feature of the hysteresis nonlinearity. The linear PI type controller, based on Ziegler–Nichols method [8], is often proposed for treating the control problems of a class of systems with the hysteresis nonlinearity. However, the linear PI type control design is only applicable with some special cases which are with slight nonlinearities. For reasons depicted above, a realizable nonlinear feedback linearization controller is proposed for treating the positioning problem of piezoelectric materials with hysteresis effects. In this investigation, the overall nano-scale positioning design of piezoelectric materials in the presence of the hysteresis nonlinearity is divided into four parts: 1. System modeling of piezoelectric materials with the hysteresis nonlinearity is introduced firstly; 2. Parameter identification method of the modelled system is expressed next; 3. The bounded input and bounded output (BIBO) stability verification for this identified model is described; 4. A nonlinear control law based on the feedback linearization method is derived to guarantee the convergence of the positioning error between the piezoelectric material’s output trajectory and the desired trajectory. At the end, conclusions are made.

## 2. Problem Formulation

### 2.1. Hysteresis Phenomenon of Piezoelectric Materials

**Figure 1:**
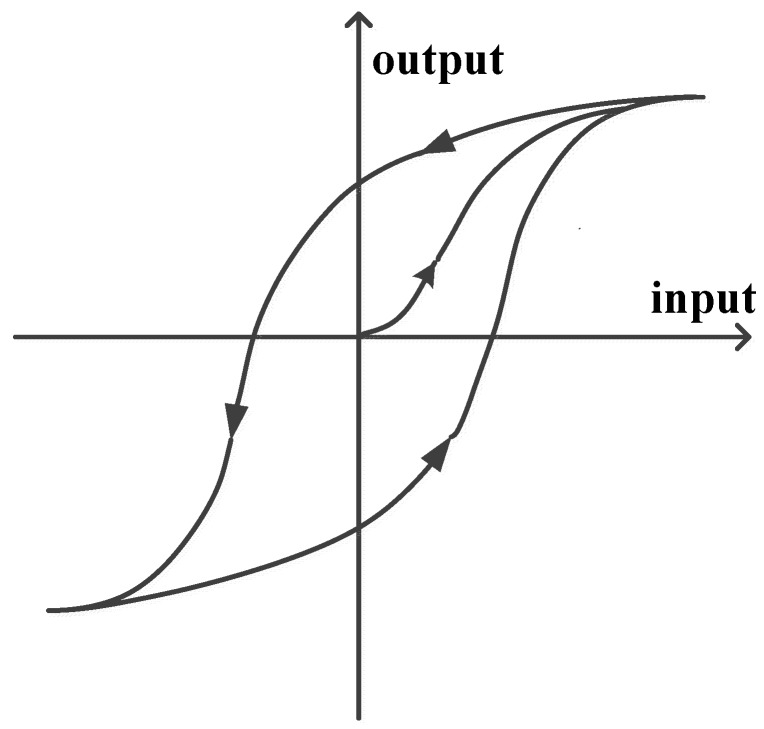
Hysteresis loop of piezoelectric materials which is a memory-like effect, and the output is independent of the input.

The nonlinear relationship between the electric field and the displacement of a piezoelectric material shows a nonlinear phenomenon called hysteresis. The output of a physical system with hysteresis is independent of the input, and as the input signal alternates between the increasing and the decreasing condition, the response curve diverges from its original path and draws a new curve. A typical hysteresis loop of systems is shown in Figure 1. Unfortunately, piezoelectric materials inherently possess this drawback and limit the application fields of this material because unacceptable positioning performances will be delivered without any compensation design. From Figure 1, the highly nonlinear feature between the input and output of piezoelectric materials is revealed. However, a systematic compensation process for eliminating the hysteresis property of systems is not yet be well-developed.

As mentioned above, the piezoelectric material possesses a strong nonlinearity between the output displacement and the input voltage. This hysteresis property causes the system oscillation and even instability. Inevitably, it massively degrades the positioning accuracy. This fatal disadvantage lets the actuator naturally have an inevitable positioning error as high as 10–15% of the desired trajectory. For effectively overcoming the effect of hysteresis and achieving high-precision positioning, a complete model for presenting the hysteresis of the piezoelectric material will first be built up. Furthermore, a nonlinear control method, which can eliminate the hysteresis and precisely control the piezoelectric material to track arbitrary given trajectories, will be developed.

### 2.2. Single-Phase-Driven Piezoelectric Material

**Figure 2:**
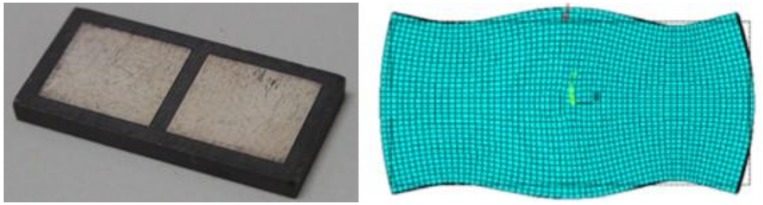
(**a**) The used piezoelectric material and (**b**) deformation simulation of the used piezoelectric material.

Existing research about developing novel signal-phase-driven piezoelectric plates is investigated in [9,10]. In these published results, one electrode is attached on the surfaces of the piezoelectric material as in Figure 2a, in the rear, and two exciter electrodes are placed on the front surface. By giving different driving voltages to the electrodes on the front surface, different degrees of deformation of the piezoelectric material can be made, as shown in Figure 2b, at nano-scale level. Based on this concept, a nano-scale positioning device can be created via controlling the deformations of piezoelectric materials.

### 2.3. Modelling for Piezoelectric Materials

In this investigation, a mathematical system formulation called “Bouc–Wen model” is adopted to explain the behavior of the piezoelectric material in Figure 2. The Bouc–Wen model is a typical semi-physical model which was initially proposed by Bouc in 1971, and then modified by Wen in 1976. For performance tests or practical simulations of a class of systems with hysteresis effect, it has the advantage of the computational simplicity and the practical similarity. From descriptions of the current literatures, the Bouc–Wen model is confirmed as a really good mathematical expression to describe the hysteresis behavior of piezoelectric materials, because it only needs a set of first order nonlinear differential equations. Based on the Bouc–Wen model formulation, relationship of the control input (*u*) and system output (*y*) can be expressed by a mathematical mapping u|→y and a set of first-order nonlinear differential equations. The generalized Bouc–Wen model for piezoelectric materials with the hysteresis effect can be described as the following:

(1) $$\{\begin{array}{c} \dot{x}=u\\ \dot{z}=D^{-1}\left(Au-\beta \left|u\right|{\left|z\right|}^{n-1}z-\gamma u{\left|z\right|}^{n}\right)\\ y=\alpha kx+\left(1-\alpha \right)Dkz \end{array}$$

Without loss of generality, this model consists of a set of first order nonlinear differential equations that associates the control input *u* with the system output *y* in a hysteretic way. By appropriately selecting a set of parameters for the model in (1), it is possible to precisely approximate the behavior of a piezoelectric material with the real hysteresis loop. If (1) is used to approximate the behavior of piezoelectric material, *u* and *y* will be the control voltage and the piezoelectric material displacement (deformation), respectively. In this nano-scale positioning design, *z* represents the hysteresis state variable, the parameters *A* and *n* are the scale of the control input and sharpness of the hysteresis loop, respectively. As to *β* and *γ*, these two system parameters control the asymmetric shape of the hysteresis loop of piezoelectric materials. A systematic selection of parameters: *n* ≥ 1, *D* > 0, *k* > 0, 0 < *α* < 1, and *β* + *γ* ≠ 0, with a proven formula will be introduced in the next section.

### 2.4. Set Up of the Practical Measurment Platform and Parameter Identification for the Used Bouc–Wen Model

**Figure 3:**
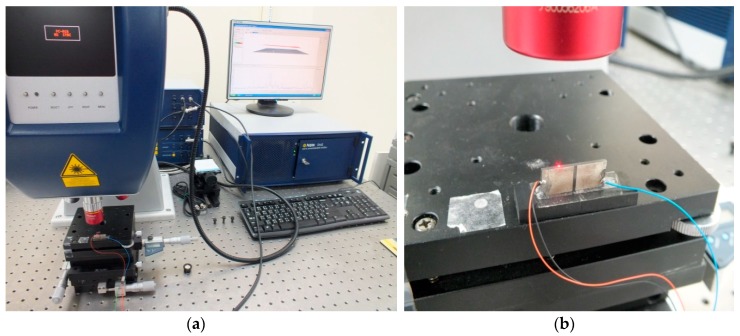
(**a**) A displacement measurement apparatus (MSN-400, Polytec, Berlin, Germany) and a control device is built up for the verification of the positioning performance of this proposed method, and (**b**) the enlarged picture of the controlled piezoelectric material in (**a**).

For the verification of the practical positioning performance, a platform with a control apparatus and a measurement device is built up as Figure 3. In this real implementation, a highly accurate displacement measurement device (1 nm accuracy, MSN-400, Polytec, Berlin, Germany) is utilized to precisely report the real-time displacement of the controlled piezoelectric material at nano-scale level.

**Figure 4:**
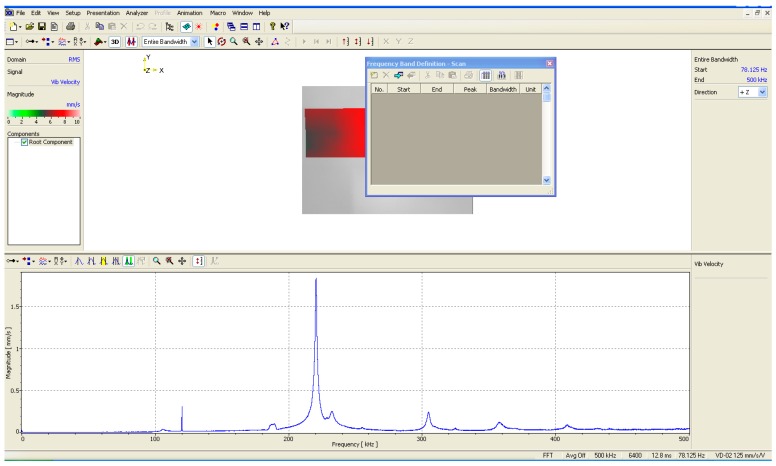
Frequency response of the piezoelectric material.

The exciting frequency which is used to effectively drive the piezoelectric material to generate the maximum displacement with a specific system mode is one of the major characteristics of the piezoelectric material. By using the displacement measurement apparatus Polytec MSN-400 to scan the displacement magnitudes of the controlled piezoelectric material with frequencies ranging from 0 to 500 KHz, the base mode frequency which maximizes the displacement of the piezoelectric material can be obtained. Figure 4 represents the searching result for a piezoelectric material, and the base mode with a frequency 215 kHz has the maximum displacement; hence, this frequency will be selected to excite the piezoelectric material.

**Figure 5:**
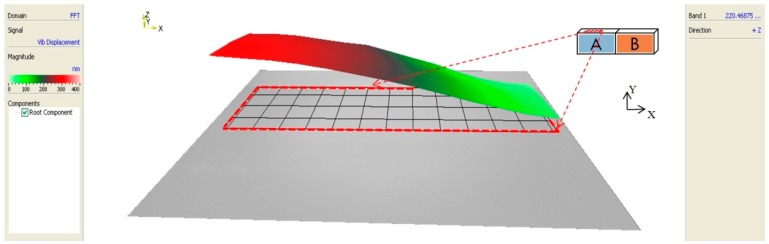
Displacement profile of a driven piezoelectric material.

From the result in Figure 4, Figure 5 presents an example that a real-time displacement of the piezoelectric material which is controlled with an input voltage 5 V, accompanying an exciting frequency of 215 kHz. Based on this example, an idea which offers the possibility to achieve a nano-scale positioning design is inspired: “Developing an algorithm to control the displacement (bright red-part) of the piezoelectric material to tracking certain trajectories at nano-scale level, a device which possesses nano-scale positioning ability can be realized”.

**Figure 6:**
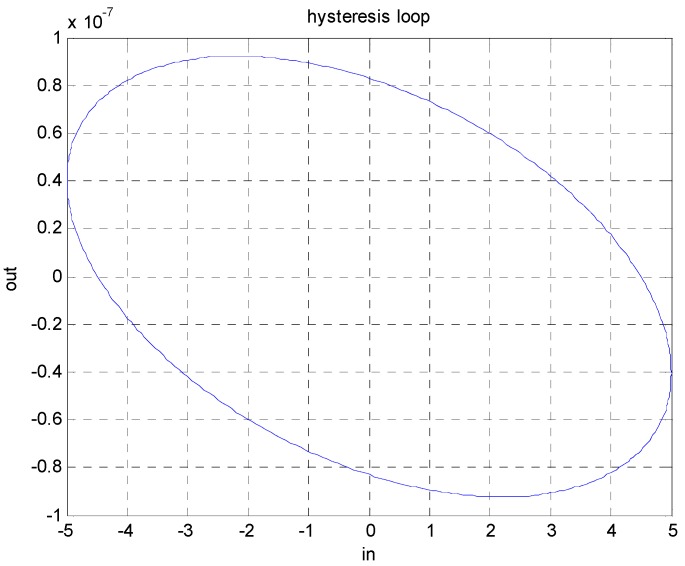
History of input voltage and output displacement for the driven piezoelectric material.

Collecting a series of input and output raw data via measuring the deformation of the driven piezoelectric material with the Polytec MSN-400, a hysteresis loop as shown in Figure 6 is illustrated. The physical meaning of this loop is that the input and output relationship of the driven piezoelectric material is not linear and is entirely a nonlinear mapping of single input and multi-output; hence, this behavior will result in a larger positioning error, and let the uncompensated piezoelectric material be not suitable for the nano-scale positioning design. For achieving an accurate positioning design, this nonlinear behavior should be modelled with a specific mathematical model and be eliminated.

In this article, the Bouc–Wen model is chosen to approximate the physical behavior of a piezoelectric material shown in Figure 6. In the Bouc–Wen model, there are seven parameters *α*, *k*, *D*, *A*, *β*, *γ*, and *n*, which should be identified simultaneously. In this study, the particle swam optimization (PSO) method [11] is given to computationally seach the optimal system parameters (*α*, *k*, *D*, *A*, *β*, *γ*, and *n*) of the Bouc–Wen model. The optimization parameters of the piezoelectric material can be identified with the following process:

1. Data collection: apply a full-range input voltage *u* (0–10 V) to drive piezoelectric material and record the corresponding output displacement *y*.
2. Model Implementation: the Bouc–Wen model is chosen for representing the behavior of the piezoelectric material.
3. Parameter searching: the PSO method model is adopted to find out the optimal parameters (*α*, *k*, *D*, *A*, *β*, *γ* and *n*) for the Bouc–Wen model.

Based on the above steps, the optimization parameters for the driven piezoelectric material are listed as Table 1.

Before developing the nano-scale positioner, stability of the Bouc–Wen model with the hysteresis effect should be checked over. Essentially, identifiable parameters of the Bouc–Wen model in (1) always affect the stability of the overall system, and should be arranged carefully. The basic stability verification for the identified Bouc–Wen model is the bounded input and bounded output (BIBO) stability property. The related concept about using BIBO stability to check the feasibility of an identified Bouc–Wen model is briefly described below.

BIBO stability of the Bouc–Wen model: Define a set: $\Omega_{\alpha ,k,D,A,\beta ,\gamma ,n}$ = {z(0)∈R such that output *y* is BIBO stable for all $C^{1}$ input signal u with fixed values of the parameters *α*, *k*, *D*, *A*, *β*, *γ*, and *n*}. Namely, z(0)∈R is a value of $\Omega_{\alpha ,k,D,A,\beta ,\gamma ,n}$ such that z(t) is bounded for any $C^{1}$ bounded input signal u with fixed values of the parameters *α*, *k*, *D*, *A*, *β*, *γ*, *n*. The set of parameters $\Omega_{A,\beta ,\gamma ,n}$ can be chosen based on the criteria in Table 2 to determine if the Bouc–Wen model is stable or not.

Based on the parameter selecting criteria in Table 2, the following two examples are given to explain how system parameters affect the Bouc–Wen model. Example 1 is an unstable Bouc–Wen model, and Example 2 is a stable case. The hysteretic loops between (*u*,*y*) will be plotted with respect to two different sets of system parameters: *α*, *k*, *D*, *A*, *β*, *γ*, and *n*.

**Figure 7:**
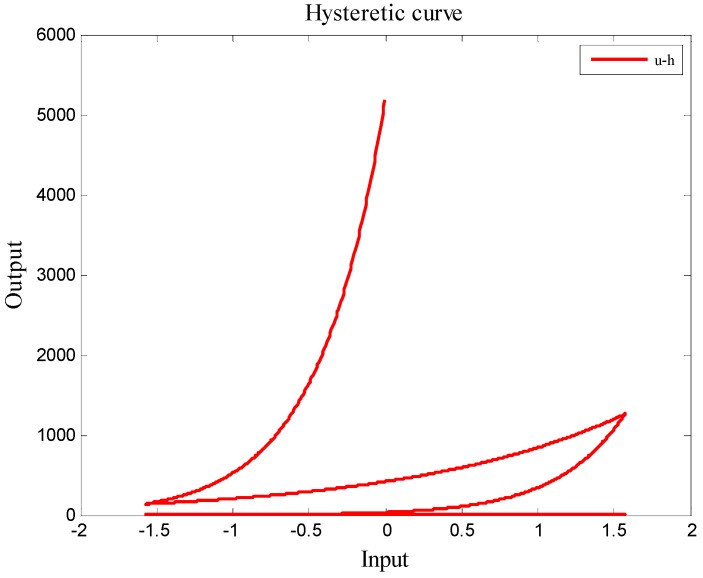
An unstable Bouc–Wen model for the piezoelectric materials.

**Example** **1.** Suppose a Bouc–Wen model is built up with parameters: D = 1, A = 1, k = 1, α = 0.5, β = 0.5, γ = −1.5, and n = 2, and the initial condition of this model is selected as z(0)=0 Furthermore, a bounded input signal u(t) = π/2sin(t) is fed into this model. From Figure 7, the simulation result exhibits an unbounded property, i.e., this model is not BIBO stable. Definitely, this is an unqualified Bouc–Wen model, and cannot be used as a suitable approximation for a piezoelectric material.

**Figure 8:**
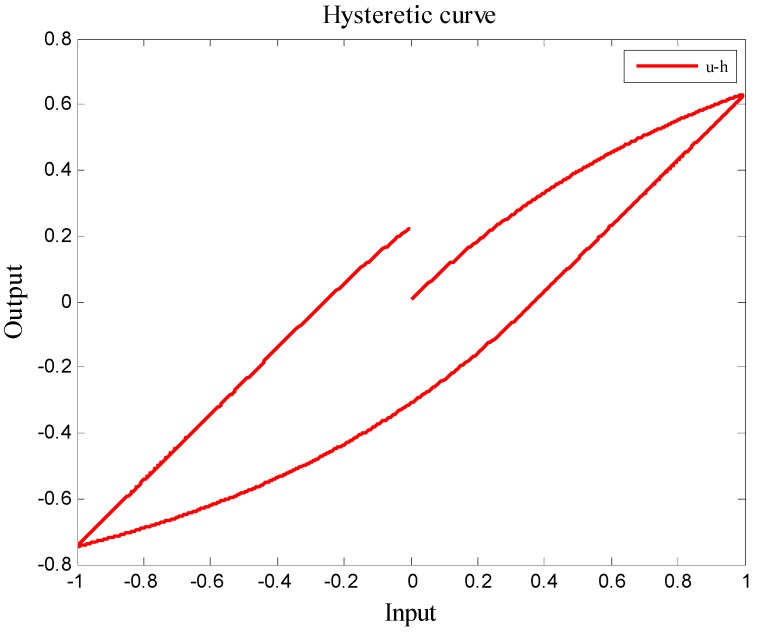
A stable Bouc–Wen model for the piezoelectric materials.

**Example** **2.** Suppose the Bouc–Wen model is built up by parameters: D = 1, A = 2, k = 1, α = 0.5, β = 0.5, γ = 0.1, and n = 1.1. Using the same initial condition and the same bounded input signal depicted in example 1, it is easy to find out that the input and output relationship is bounded from Figure 8; hence, this Bouc–Wen model is BIBO stable, and this model is a qualified approach for the piezoelectric material.

Using the above criterion, a stable model can be theoretically found, and it will be utilized to develop a nonlinear control law for the positioning problem of piezoelectric materials.

## 3. Nano-Scale Positioning Design

The design objective is to find a nonlinear control law to drive the piezoelectric material to track desired trajectories at nano-scale level with as small as possible positioning errors.

Define the positioning error as

(2) $$e(t)=y(t)-y_{m}(t)$$

From the above description, the control law *u*(*t*) in (1) should be specified such that the hysteresis effect on the positioning error *e*(*t*) must be attenuated as much as possible; hence, the performance index for this positioning problem can be formulated as

(3) $$\underset{u(t)\in L_{2}[0,t_{f}]}{\min}\int_{0}^{t_{f}}e{\left(t\right)}^{2}dt$$

where the final time *t_f_* > 0.

Or

(4) $$\underset{t\to \infty }{\lim}e(t)=0$$

For the purposes of practical realization and reducing calculation complexity, a nonlinear control methodology with a really simple form is required. For achieving the above design objective with a control structure with a low calculation power, the easy-to-implement nonlinear feedback linearization method will be utilized. Let us denote the positioning error *e* as the system output, and *u* is the control input. A nonlinear control law which converges the positioning error *e* to zero will be obtained by the following steps.

Step 1: Differentiating positioning error equation in (2) with respect to time, and we then have

(5) $$\begin{array}{ll} \dot{e} & =\dot{y}-{\dot{y}}_{m}\\ & =\alpha ku+\left(1-\alpha \right)k\left(Au-\beta \left|u\right|{\left|z\right|}^{n-1}z-\gamma u{\left|z\right|}^{n}\right)-{\dot{y}}_{m} \end{array}$$

Obviously, the control message *u* can be found from (5).

Step 2: By selecting the nonlinear control law *u* as

(6) $$u=\frac{1}{g(z)}\left\{{\dot{y}}_{m}-\lambda e\right\}$$

where the time-varying control parameter $g\left(z\right)=\frac{-1}{\alpha k}\left\{\left(1-\alpha \right)k\left(A-\beta \mathrm{sgn}\left(u\right){\left|z\right|}^{n-1}z-\gamma {\left|z\right|}^{n}\right)\right\}$, and *λ* is a virtual control law. 

Substituting the control law (6) into (5) yields the following positioning error dynamics

(7) $$\dot{e}+\lambda e=0$$

For the convergence of the positioning error, the coefficient *λ* > 0 in (6) must be suitably selected to let the characteristic equation in (7) be a Hurwitz polynomial (i.e., roots of (7) are all in the left-half complex plane). This implies that the overall positioning error dynamics are asymptotically stable, and positioning error *e* will converge to zero asymptotically, with a convergence rate that depends on the choice of *λ*.

**Theorem** **1.** *For the positioning problem of the piezoelectric material in (1) and (3), the following nonlinear controller is designed to drive the piezoelectric material to precisely track the desired trajectory and eliminate the hysteresis effect.*

(8) $$u=\frac{1}{g(z)}\left\{{\dot{y}}_{m}-\lambda e\right\}$$

Based on this proposed control law, the positioning performance index in (3) can be guaranteed, and the positioning error *e* can be proven to converge to zero asymptotically. 

## 4. Verification of the Positioning Performance

**Figure 9:**
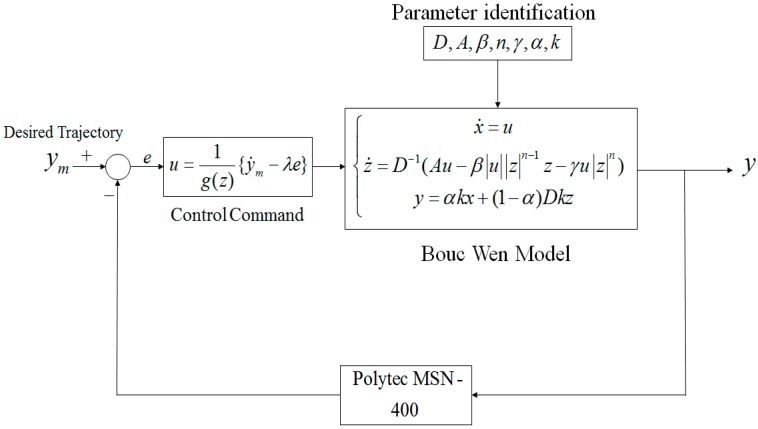
The closed-loop nano-scale positioning design for piezoelectric materials.

Flow chart of the overall nano-scale positioning design is exhibited in Figure 9. In Figure 9, the controlled piezoelectric material is modelled by the Bouc–Wen model first, and the PSO method is then offline used to identify parameters of Bouc–Wen model based on the measured raw data pairs (*u*, *y*). Based on the measurement output *y*, a nonlinear positioing design *u* in (6) is constructed.

For verifying the positioning performances of the proposed method, two typical trajectories 1. A square trajectory, and 2. A periodic trapezoidal trajectory, are chosen as the desired trajectories.

**Trajectory** **1:** A square trajectory with positive magnitudes.

**Figure 10:**
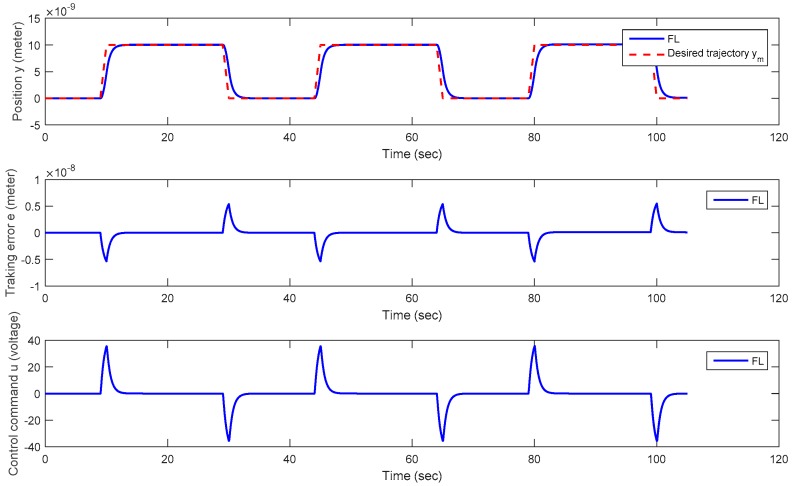
Plots of the positioning history *y*, positioning error *e,* and control command *u* versus time for Trajectory 1 (a square trajectory).

In this testing scenario, a square trajectory with all values within a nano-scale range *y_m_* ∈ [0 (nm), 10 (nm)] is used. The positioning results for this test are shown in Figure 10. From the positioning history exhibited in Figure 10, and the comparison of the output trajectory *y* and the desired trajectory *y_m_*, it is easy to realize that this proposed method offers really promising positioning capability for the piezoelectric material at nano-scale level, because no overshoot exists in the positioning history. Furthermore, an asymptotically convergent property can be found in the history of the positioning error. A similar convergent character can be obtained for the control command *u* as well.

**Trajectory** **2:** A periodic trapezoidal trajectory with positive and negative magnitudes.

**Figure 11:**
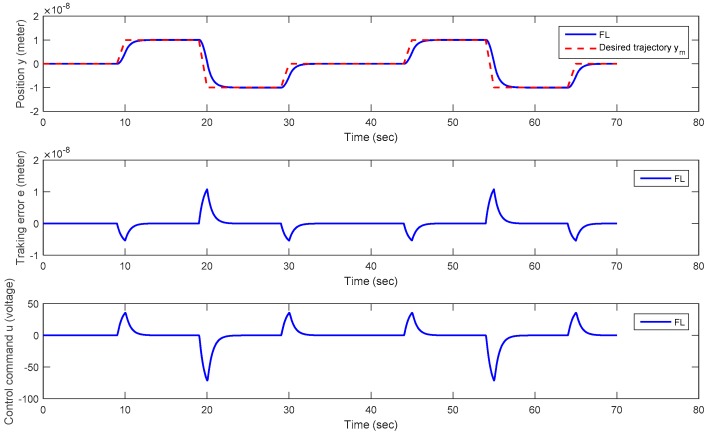
Plots of the positioning history *y*, positioning error *e*, and control command *u* versus time for Trajectory 2 (a periodic trapezoidal Trajectory).

In this testing scenario, a periodic trapezoidal trajectory with positive and negative values within a nano-scale range *y_m_* ∈ [−100 (nm), 100 (nm)] is adopted. From Figure 11, the output *y* of the piezoelectric material precisely follows the desired periodic trapezoidal trajectory *y_m_* without overshoots. From Figure 11, both the positioning error *e* and the applied control voltage *u* converge to zero with a 2 seconds transient time. In practical applications, this positioning response is acceptable. Suppose that a faster response is required, it can be improved by selecting a larger virtual control law *λ* in (5). Nevertheless, the larger *λ* is, the larger control voltage *u* is.

From the testing results of the above scenarios, a satisfactory positioning performance can be obtained for piezoelectric materials at nano-scale level via use of the proposed method. This proposed control method can be further extended to the situation which modeling uncertainties and external disturbances of piezoelectric materials are considered.

## 5. Conclusions

A nonlinear positioning design with an ultrahigh accuracy is successfully developed for piezoelectric materials after mitigating the hysteresis nonlinearity. This proposed method lets the controlled piezoelectric material be capable of precisely tracking certain predefined trajectories, such as the square and the periodic trapezoidal trajectory at the nano-scale level. In this investigation, the positioning error can be proven to converge to zero via minimizing the positioning performance index. Besides, this proposed method has a really simple control structure, and can be easily implemented for improving the positioning accuracy of piezoelectric materials in the presence of the hysteresis effect that is generally hard to deal with. From the real tests, this proposed nano-scale positioning design is surperior to the conventional design, no matter in calculation power consumption or in the convergence of positioning errors; hence, this proposed method provides opportunities for piezoelectric materials to be widely used in nano-scale positioning applications.

## Figures and Tables

**Table 1:** Identified parameters for the driven piezoelectric material with a Bouc–Wen model.

Parameter	Value	Unit
*n*	1	(N/A)
*A*	1	(V/m)
*k*	1.9567 × 10^5^	(N/m)
*D*	1.7339 × 10^−6^	(m/V)
*α*	0.3575	(N/A)
*β*	0.0364	(N/A)
*γ*	0.0272	(N/A)

**Table 2:** Classification of BIBO stable Bouc–Wen model.

Case	Condition	Ω	Upper Bound on |z(t)|	Class
*A* > 0	*β* + *γ* > 0 and *β* − *γ* ≥ 0	R	max$\left(\left|z\left(0\right)\right|,z_{0}\right)$	I
	*β* − *γ* < 0 and *β* ≥ 0	[−$z_{1},z_{1}$]	max$\left(\left|z\left(0\right)\right|,z_{0}\right)$	II
*A* < 0	*β* − *γ* > 0 and *β* + *γ* ≥ 0	R	max$\left(\left|z\left(0\right)\right|,z_{1}\right)$	III
	*β* + *γ* < 0 and *β ≥* 0	[−$z_{0},z_{0}$]	max$\left(\left|z\left(0\right)\right|,z_{1}\right)$	IV
*A* = 0	*β* + *γ* > 0 and *β* − *γ* ≥ 0	R	|z(0)|	V

## References

[1] Jiles D.C., Atherton D.L. (1986). Theory of ferromagnetic hysteresis. J. Magn. Mater..

[2] Brokate M., Sprekels J. (1996). Hysteresis and Phase Transitions.

[3] Iyer R.V., Tan X., Krishnaprasad P.S. (2005). Approximate inversion of the preisach hysteresis operator with application to control of smart actuators. IEEE Trans. Autom. Control.

[4] Webb G.V., Lagoudas D.C., Kurdila A.J. (1998). Hysteresis modeling of SMA actuators for control application. J. Int. Mater. Syst. Struct..

[5] Kuhnen K. (2003). Modeling, identification and compensation of complex hysteresis nonlinearities, a modified Prandtl-Ishlinskii approach. Eur. J. Control.

[6] Ismail M., Ikhouane F., Rodellar J. (2009). The hysteresis Bouc-Wen model, a survey. Arch. Comput. Methods Eng..

[7] Ikhouane F., Mañosa V., Rodellar J. (2007). Dynamic properties of the hysteretic Bouc-Wen model. Syst. Control Lett..

[8] Xu H.G., Ono T., Esashi M. (2006). Precise motion control of a nanopositioning PZT microstage using integrated capacitive displacement sensors. J. Micromech. Microeng..

[9] Otokawa K., Takemura K., Maeno T. (2007). A Multi-Degree of freedom ultrasonic motor using single-phase-driven vibrators. Trans. Jpn. Soc. Mech. Eng. Part C (Nihon Kikai Gakkai Ronbunshu C Hen).

[10] Vyshnevskyy O., Kovalev S., Wischnewskiy W. (2005). A novel, single-mode piezoelectric plate actuator for ultrasonic linear motors. IEEE Trans. Ultrason. Ferroelectr. Freq. Control.

[11] Kennedy J., Eberhart R. Particle swarm optimization. Proceedings of the IEEE International Conference on Neural Networks.

